# Research on the Influence of Carbon Sources and Buffer Layers on the Homogeneous Epitaxial Growth of 4H-SiC

**DOI:** 10.3390/mi15050600

**Published:** 2024-04-29

**Authors:** Weilong Yuan, Yicheng Pei, Yunkai Li, Ning Guo, Xiuhai Zhang, Xingfang Liu

**Affiliations:** 1 Key Laboratory of Semiconductor Materials Science, Institute of Semiconductors, Chinese Academy of Sciences, Beijing 100083, China; yuanweilong@semi.ac.cn (W.Y.); peiyicheng@semi.ac.cn (Y.P.); liyunkai@semi.ac.cn (Y.L.); guoning@semi.ac.cn (N.G.); 2 School of Resources, Environment and Materials, Guangxi University, Nanning 530004, China; 3 College of Materials Science and Opto-Electronic Technology, University of Chinese Academy of Sciences, Beijing 100049, China; 4 Beijing Key Laboratory of Low Dimensional Semiconductor Materials and Devices, Beijing 100083, China

**Keywords:** 4H-SiC homoepitaxial layer, Chemical vapor deposition (CVD), carbon source, buffer layer, growth pressure

## Abstract

In this study, a 4H-SiC homoepitaxial layer was grown on a 150 mm 4° off-axis substrate using a horizontal hot wall chemical vapor deposition reactor. Comparing C_3_H_8_ and C_2_H_4_ as C sources, the sample grown with C_2_H_4_ exhibited a slower growth rate and lower doping concentration, but superior uniformity and surface roughness compared to the C_3_H_8_-grown sample. Hence, C_2_H_4_ is deemed more suitable for commercial epitaxial wafer growth. Increasing growth pressure led to decreased growth rate, worsened thickness uniformity, reduced doping concentration, deteriorated uniformity, and initially improved and then worsened surface roughness. Optimal growth quality was observed at a lower growth pressure of 40 Torr. Furthermore, the impact of buffer layer growth on epitaxial quality varied significantly based on different C/Si ratios, emphasizing the importance of selecting the appropriate conditions for subsequent device manufacturing.

## 1. Introduction

The limitations of traditional Si-based power devices in advanced applications are becoming apparent due to low conduction voltage drops and limited high-frequency switching capabilities [1,2], becoming significant factors hindering the progress of modern electronic equipment. On the other hand, SiC devices offer a new solution due to their durable nature and exceptional performance in extreme conditions [3,4]. This durability is not only evident in their ability to withstand high temperatures and electrical loads but also in their stability in high-radiation environments, a feat that Si devices struggle to match [5,6,7]. The low power consumption of SiC devices is particularly crucial in today’s electronic devices, given the increasing demand for power efficiency and heat dissipation [8]. This characteristic allows SiC devices to generate less heat under the same operating conditions, thereby alleviating the strain on heat dissipation systems. Consequently, this not only facilitates equipment miniaturization but also reduces operating and maintenance costs, while enhancing equipment reliability and longevity by lowering cooling system requirements. As manufacturing technology advances and economies of scale are realized, the cost of SiC devices gradually decreases, leading to expanded applications across various industries [9,10]. In the long term, SiC technology is poised to revolutionize key fields such as energy efficiency, power electronics, transportation, and industrial automation, offering more efficient and reliable technical solutions and driving industries toward a more sustainable and environmentally friendly future [11].

The production of SiC epitaxial wafers is crucial for manufacturing SiC devices [12]. As the complexity and diversity of the working environment continue to increase, the quality standards for SiC devices become more stringent. SiC devices must exhibit increased reliability, stability, and durability to withstand various extreme conditions and high-intensity workloads [13]. The key to achieving these objectives lies in enhancing the quality of the SiC epitaxial layer, as its quality significantly impacts the overall performance and longevity of the device [14,15]. Consequently, elevated demands have been placed on improving and controlling the growth rate, doping concentration, and surface quality of the epitaxial layer. Chemical vapor deposition (CVD) is a widely used technique for depositing thin films on a substrate through chemical reactions of vapor precursors at high temperatures [16]. By adjusting process parameters, the film’s structure can be modified [17,18]. In comparison to other growth methods, CVD offers precise control over the thickness, impurity doping, and uniformity of the epitaxial layer [19]. As a result, the 4H-SiC homoepitaxial layer produced using this method exhibits superior quality and can be directly utilized in the fabrication of SiC devices.

Different growth process parameters significantly influence epitaxial quality. Various carbon sources exhibit distinct crystal structures, purity levels, and reactivity, directly impacting the crystal structure and purity of the epitaxial layer [20,21]. High-purity carbon sources can diminish impurities and defects in the epitaxial layer, thereby enhancing crystal quality and electrical performance [22]. Greater reactivity can accelerate epitaxial growth rates but may also elevate roughness and defect density [23]. Moreover, a buffer layer can mitigate the lattice mismatch and stress between the substrate and epitaxial layer, enhancing quality and uniformity [24,25]. By optimizing buffer layer growth conditions like temperature, thickness, and composition, improvements in crystal structure, surface morphology, and doping characteristics of the epitaxial layer can be achieved [26,27]. The buffer layer also acts as a barrier against impurities and defects, reducing defect density and enhancing overall performance. Prior studies have primarily focused on the impact of C/Si ratio, growth temperature, Si/H ratio, and Si source selection on crystal form and quality of epitaxial layer [28,29,30]. This article delves into the influence of C source selection, buffer layer growth, and changes in growth pressure on epitaxial quality. The findings indicate that optimizing epitaxy quality through process parameter adjustments necessitates a holistic consideration of the comprehensive impact post-change, rather than focusing solely on individual factors.

## 2. Materials and Methods

Growth experiments were conducted in a horizontal hot-wall chemical vapor deposition system using a 150 mm 4° off-axis 4H-SiC substrate [31]. The experiments were carried out under standard process conditions of 1570 °C and 40 Torr pressure. Trichlorosilane (TCS) was utilized as a silicon source with a flow rate of 50 sccm. H_2_ was utilized as the dilution, carrier, and etching gas at a flow rate of 100 slm. Initially, propane (C_3_H_8_) and ethylene (C_2_H_4_) were chosen to assess the influence of the carbon source on the quality of the epitaxial layer, while maintaining a C/Si ratio of 1. Subsequently, pressure comparison experiments were conducted with a C/Si ratio held at 0.72 and pressures of 40 Torr, 50 Torr, 60 Torr, and 70 Torr. The buffer layer was then grown for 4 min with a C/Si ratio of 1 and an N_2_ flow rate of 50 sccm, followed by standard epitaxial growth lasting for 30 min. The epitaxial layer thickness on all wafers was determined using a Fourier transform infrared spectrometer (FTIR, Nicolet IS50, Thermo Fisher, Waltham, MA, USA), with 29 measurement points selected on the wafer surface, including 8 points at the center and 4 points located 5 mm from the edge. Further analysis will focus on points 1 to 15. Doping concentration was assessed through capacitance-voltage (CV, MCV-530L Semilab, Budapest, Hungary) measurements using Hg Schottky contacts, with a 5 mm edge offset during measurement. Surface roughness was evaluated using an atomic force microscope (AFM, AFM Dimension Icon, Bruker, Billerica, MA, USA) [32] on a 10 μm × 10 μm area.

## 3. Results and Discussion

### 3.1. Effect of C Source

#### 3.1.1. Effect of C Source on Growth Rate and Uniformity

**Figure 1:**
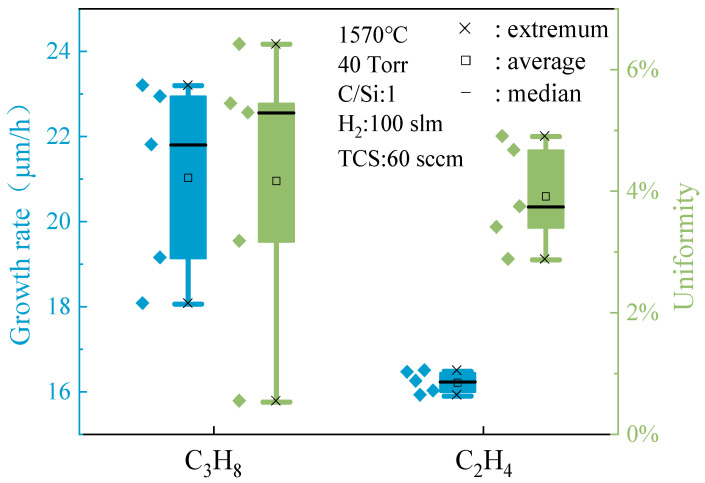
Epitaxial growth rate and thickness uniformity of C sources.

Figure 1 illustrates that utilizing C_3_H_8_ as the carbon source for growth results in a significantly higher growth rate compared to using C_2_H_4_. This indicates the superior efficiency of C_3_H_8_ in promoting faster epitaxial layer growth under similar conditions. However, rapid growth does not guarantee uniform growth quality. Further analysis of the growth rate distribution of individual samples revealed distinct differences. Samples grown with C_3_H_8_ exhibit a wide dispersion in growth rate, indicating significant fluctuations among samples. Conversely, samples grown with C_2_H_4_ show a more concentrated growth rate distribution with smaller fluctuations, suggesting greater stability. While the minimum surface thickness uniformity value of C_3_H_8_-grown samples may be low, indicating some uniformity in certain areas, the distribution range is wide, resulting in overall sample surface thickness variation and suboptimal uniformity. On the other hand, although the overall surface thickness uniformity value of C_2_H_4_-grown samples is slightly higher, implying a slightly larger average thickness difference, the distribution is more compact with a smaller variation range. This highlights that using C_2_H_4_ as the carbon source can maintain a relatively consistent level of surface thickness quality across samples, demonstrating better uniformity.

#### 3.1.2. Effect of C Source on Doping Concentration and Uniformity

**Figure 2:**
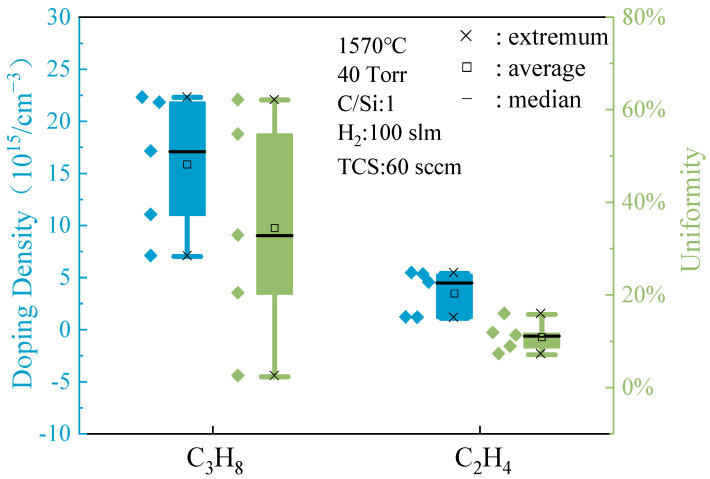
Doping concentration and uniformity of C source epitaxial layers.

Figure 2 illustrates the distinct impact of various C sources on the doping concentration and uniformity of the epitaxial layer. The data clearly indicate that the doping concentration in the epitaxial layer produced using C_2_H_4_ is generally lower compared to that grown with C_3_H_8_, whether examining a single sample or the average across all samples. This discrepancy can be attributed to the differing chemical properties of C_2_H_4_. C_2_H_4_, being more chemically active than C_3_H_8_, readily decomposes at elevated temperatures to create a C-rich environment [33]. During the growth process of 4H-SiC, N atoms and C atoms have the same lattice site occupancy, which leads to a competition effect between them. In a Si-rich environment, N atoms can effectively replace the lattice positions of C atoms, thereby significantly improving the N_2_ doping efficiency. This environment aids in minimizing impurity or dopant introduction during growth, leading to reduced doping concentration. Consequently, C_2_H_4_ demonstrates superior efficacy in regulating doping concentration. In terms of uniformity, the epitaxial layer from the C_2_H_4_-grown sample also displays commendable performance. Analysis of samples grown with different C sources reveals that the C_2_H_4_-grown sample exhibits a more concentrated distribution of uniformity values, indicating minor variations in doping concentrations across different regions. Moreover, the values are generally low, suggesting high overall doping concentration uniformity. Conversely, the uniformity of the C_3_H_8_-grown sample appears slightly less favorable. Therefore, C_2_H_4_ as a C source not only excels in controlling doping concentration in the epitaxial layer but also demonstrates exceptional uniformity in maintaining doping concentration.

#### 3.1.3. Effect of C Source on Roughness

**Figure 3:**
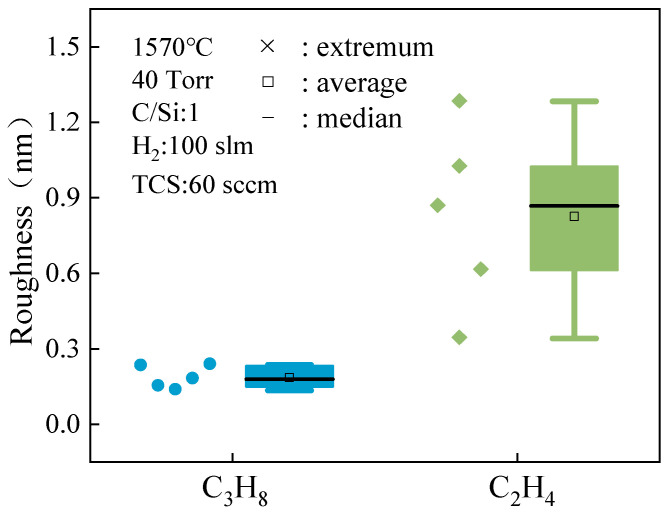
The surface roughness of different C source epitaxial layers.

However, when turning to observations of the surface morphology of 4H-SiC, the situation changes. Surface topography is an important factor in evaluating material quality and device performance, especially in the post-production stage of devices, such as the formation of ohmic contacts. Figure 3 illustrates the roughness comparison in epitaxial layers grown using different C sources.

**Figure 4:**
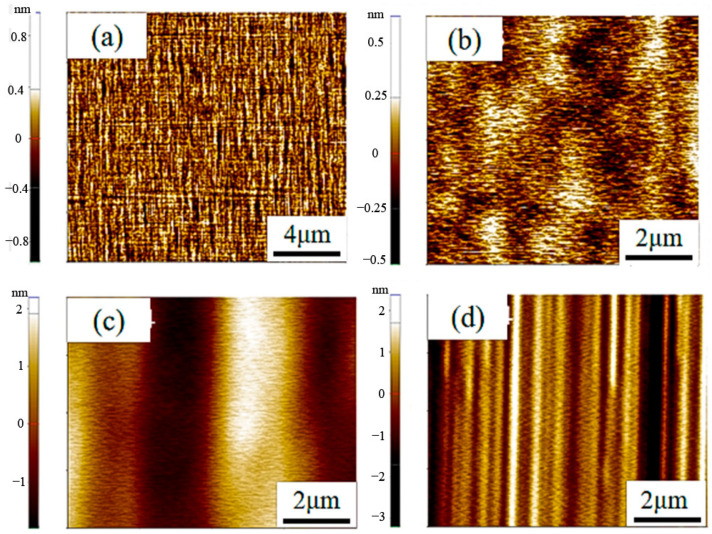
AFM images of epitaxial layers grown by different C sources: (**a**,**b**) C_3_H_8_; (**c**,**d**) C_2_H_4_.

The data clearly indicate that epitaxial layers grown with C_3_H_8_ exhibit extremely low surface roughness, measuring less than 0.25 nm and showcasing a remarkably smooth surface. This low roughness surface is advantageous for facilitating good ohmic contact formation and enhancing device performance. Conversely, epitaxial layers grown with C_2_H_4_ display relatively high surface roughness, with significant variation among different samples. This elevated roughness could result in subpar ohmic contact effects and compromise overall device performance. The AFM images in Figure 4 further support these findings, showing more undulations and irregular structures on samples grown with C_2_H_4_ compared to the relatively flat surface of samples grown with C_3_H_8_. It is hypothesized that the difference in surface quality may be attributed to the differing chemical reactivity of C_2_H_4_ and C_3_H_8_. Specifically, the increased likelihood of decomposition and reaction of C_2_H_4_ during high-temperature growth could contribute to surface instability and unevenness. Due to its more stable chemical properties, C_3_H_8_ may be more conducive to forming a uniform and smooth surface during the growth process. Therefore, C_3_H_8_ may be a more suitable choice in device applications that pursue low surface roughness and good ohmic contact effects.

### 3.2. Effect of Growth Pressure

#### 3.2.1. Effect of Growth Pressure on Growth Rate and Uniformity

**Figure 5:**
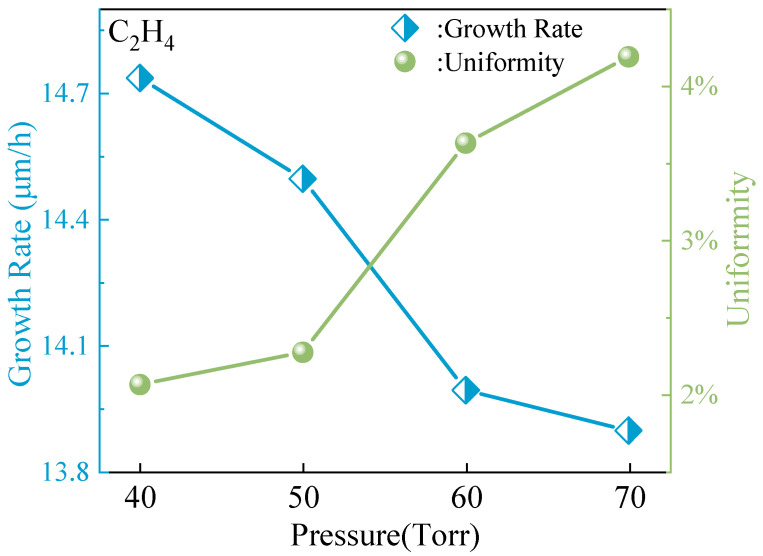
Epitaxial growth rate and thickness uniformity under different pressures.

The influence of growth pressure on SiC epitaxy manifests in changing the crystal quality, thickness, and doping concentration of the epitaxial layer. Therefore, selecting the appropriate growth pressure based on specific process conditions and device requirements is crucial to achieving high-quality epitaxial layers and optimal device performance [34]. Further research is needed to better understand and control the impact of growth pressure on SiC epitaxy. As shown in Figure 5, the changes in the epitaxial growth rate and thickness uniformity under different pressure conditions are depicted in detail. It can be clearly seen that as the pressure in the growth chamber gradually increases, the epitaxial growth rate gradually shows a decreasing trend. This phenomenon clearly shows that the increase in pressure has a significant negative impact on the growth rate of the epitaxial layer. At the same time, as the pressure continues to rise, the uniformity of the thickness of the epitaxial layer gradually shows a deteriorating trend. These further reveal that excessively high-pressure conditions may cause the growth process of the epitaxial layer to become more complex and uneven, resulting in significant differences in the thickness of the epitaxial layer in different regions. The pressure’s influence on the chemical state of the substrate surface and adsorption kinetics must be considered when analyzing this phenomenon. Lower pressure conditions create an ideal substrate surface state, facilitating the adsorption of growth reactants and promoting epitaxial layer growth. However, as pressure rises, the increased concentration of gas molecules can alter the substrate surface state, leading to easier desorption of reactants. This desorption directly impacts growth rate and thickness uniformity, making the process more complex and challenging to regulate.

#### 3.2.2. Effect of Growth Pressure on Doping Concentration and Uniformity

**Figure 6:**
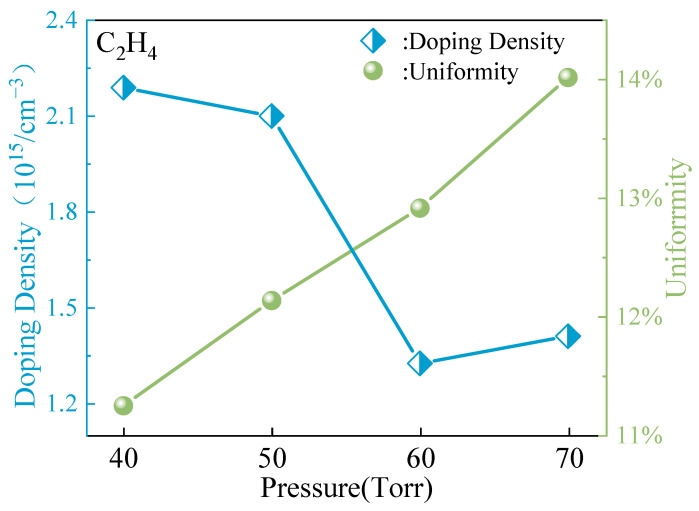
Doping concentration and uniformity of epitaxial growth under different pressures.

Upon closer examination of Figure 6, it is evident that as the growth chamber pressure gradually increases, the doping concentration of the epitaxial layer exhibits a decreasing trend. This observation indicates that the pressure not only impacts the growth rate and thickness uniformity of the epitaxial layer but also significantly influences the doping concentration. Additionally, with increasing pressure, the uniformity of the doping concentration in the epitaxial layer diminishes. This suggests that excessively high-pressure conditions can complicate and disrupt the growth process of the epitaxial layer, leading to substantial variations in doping concentrations across different regions. This phenomenon is likely linked to the influence of pressure on material transport and reaction kinetics during growth. Higher pressures may elevate the frequency of gas molecule collisions, yet this escalation does not necessarily facilitate uniform growth of the epitaxial layer. Conversely, excessive pressure could result in uneven reactant coverage on the substrate surface or heightened defect density within the epitaxial layer, thereby impacting the doping concentration and uniformity of the epitaxial layer.

#### 3.2.3. Effect of Growth Pressure on Roughness

**Figure 7:**
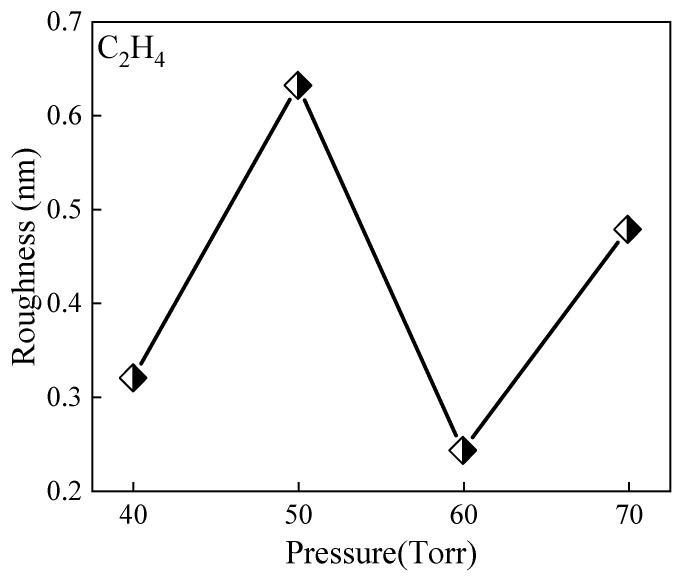
Surface roughness of epitaxial growth under different pressures.

Figure 7 illustrates the surface roughness data of epitaxial growth under varying pressures. The figure reveals a nuanced and intricate trend in surface roughness as growth pressure is manipulated. Initially, at low pressures, the epitaxial layer displays relatively high surface roughness. This phenomenon can be attributed to sluggish material transport and reaction kinetics, leading to non-uniform deposition and surface irregularities.

As the pressure is increased, a notable improvement in surface roughness is observed. This improvement likely stems from the intensified transport of precursor species and enhanced reaction kinetics, facilitating smoother and more uniform epitaxial growth. The increased pressure promotes efficient precursor delivery to the substrate surface, enabling the formation of a well-defined crystalline structure with reduced surface roughness.

However, beyond a certain pressure threshold, a reversal in the trend of surface roughness is noted. Despite the initial improvements, further pressure increases lead to a resurgence of surface roughness. This phenomenon may arise from several factors, including excessive pressure causing non-uniform reactant coverage on the substrate surface or an escalation in defect density within the epitaxial layer.

The resurgence of surface roughness at higher pressures underscores the delicate balance required in epitaxial growth conditions. While moderate pressure enhancements can enhance growth kinetics and improve surface morphology, excessive pressures can disrupt the growth process, resulting in compromised material quality and increased surface irregularities [35].

It should be noted that due to the small number of samples, the influence of sporadic factors on the surface roughness data cannot be completely ruled out. With a limited number of samples, chance factors may cause fluctuations in the data, thus affecting the accurate judgment of the overall trend. Therefore, in future research, it is necessary to further increase the number of samples to improve the reliability and accuracy of the data and thereby gain a more comprehensive understanding of the laws of epitaxial growth under different pressure conditions.

### 3.3. Effect of Buffer Layer Growth

#### 3.3.1. Effect of Buffer Layer Growth on Growth Rate and Uniformity

Buffer layer growth has a significant impact on the 4H-SiC homoepitaxial layer growth. The buffer layer can reduce the lattice mismatch and stress between the substrate and the epitaxial layer, thereby improving the quality and uniformity of the epitaxial layer [36]. By optimizing the growth conditions of the buffer layer, such as temperature, thickness, and composition, the crystal structure, surface morphology, and doping characteristics of the epitaxial layer can be improved [37]. The buffer layer can also serve as a barrier layer for impurities and defects, reducing the defect density in the epitaxial layer and improving the overall performance of the epitaxial layer. Therefore, buffer layer growth is an indispensable key link in the growth process of the 4H-SiC homoepitaxial layer [38].

**Figure 8:**
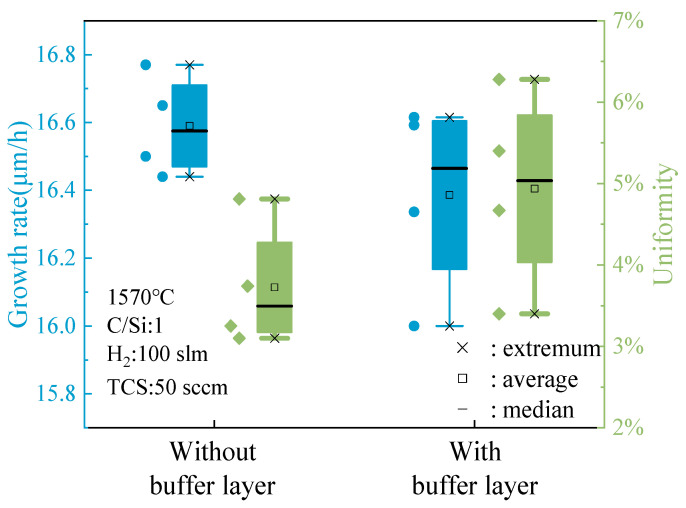
The effect of the buffer layer on the growth rate and thickness uniformity of the epitaxial layer when C/Si = 1.

When the C/Si ratio is 1, as depicted in Figure 8, a detailed analysis of the data presented in the box plot reveals a notable disparity in the epitaxial layer growth rate between samples with and without a buffer layer. Specifically, the sample grown with a buffer layer exhibited significant numerical fluctuations in growth rate, indicating more pronounced changes during the growth process and potential instability. On the other hand, the sample without a buffer layer demonstrated a higher overall growth rate with relatively minor fluctuations, showcasing more stable and predictable growth behavior. The absence of a buffer layer simplifies the growth process, minimizing interfacial complexities and promoting smoother epitaxial layer formation. This consistent growth rate suggests a more controlled deposition environment, facilitating uniform material deposition and enhanced crystal quality.

In terms of epitaxial layer thickness uniformity, the sample without a buffer layer exhibited clear advantages. Comparing the box plots of the two sample groups revealed that the thickness uniformity fluctuation in the sample without a buffer layer was notably superior to that of the sample with a buffer layer. This indicates that samples lacking a buffer layer maintain a more consistent thickness throughout growth, thereby enhancing the quality and reliability of the epitaxial layer.

The enhanced thickness uniformity observed in samples without a buffer layer can be attributed to the simplified growth dynamics and reduced likelihood of interface-induced perturbations. Without the buffer layer acting as an additional growth interface, the epitaxial layer can grow more smoothly, leading to a more uniform thickness distribution across the substrate surface. This uniformity is crucial for ensuring consistent device performance and reliability in semiconductor applications.

**Figure 9:**
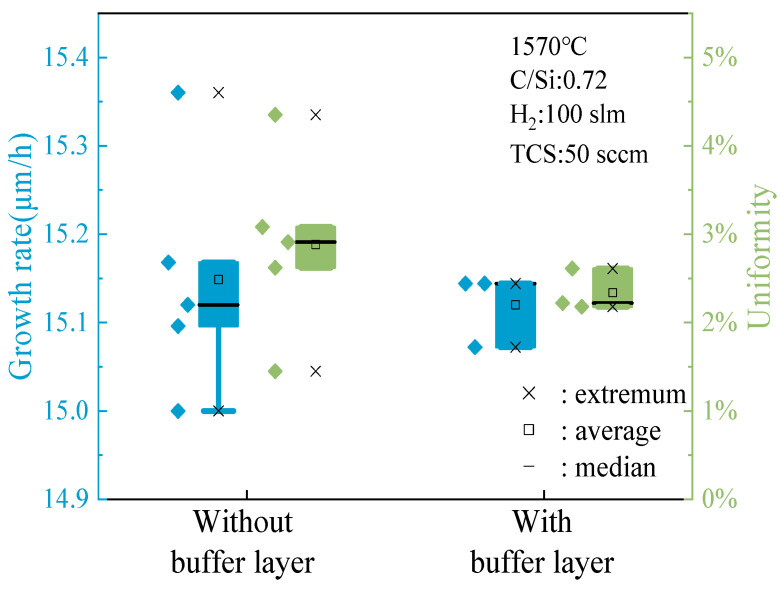
The effect of the buffer layer on the growth rate and thickness uniformity of the epitaxial layer when C/Si = 0.72.

To assess the universality and applicability of the findings, a sample with a C/Si ratio of 0.72 was specifically chosen for the buffer layer growth experiment, followed by a detailed analysis. Figure 9 illustrates that at a C/Si ratio of 0.72, distinct observations are made compared to a ratio of 1. In instances without a buffer layer, sporadic outliers in growth rate were noted, likely stemming from unstable factors during the growth phase. Conversely, samples grown with a buffer layer displayed consistent growth patterns without such outliers. While the majority of data distributions between the two scenarios were similar and concentrated, the sample with a buffer layer exhibited a more tightly clustered value distribution, indicating greater uniformity in epitaxial layer thickness. This uniformity is beneficial for enhancing the overall quality and performance of the epitaxial layers.

By comparing the buffer layer growth experiments under two different C/Si ratio conditions, it is not difficult to find whether the growth of the buffer layer has a positive effect on the growth of the epitaxial layer is closely related to the C/Si ratio during the epitaxial growth process. This may be because different C/Si ratios will affect chemical reactions and material transport during the growth process, thereby affecting the interaction and growth characteristics between the buffer layer and the epitaxial layer.

#### 3.3.2. Effect of Buffer Layer Growth on Roughness

**Figure 10:**
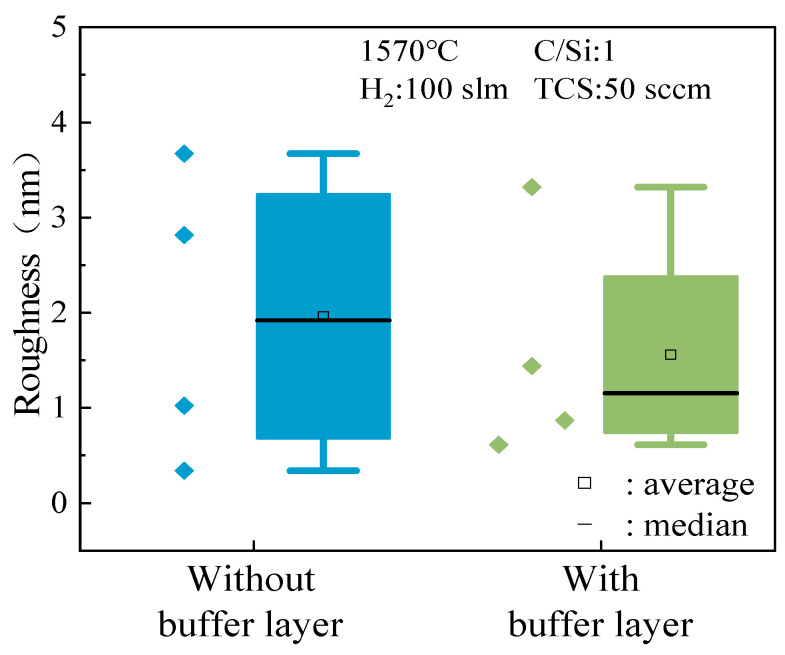
The effect of the buffer layer on the roughness of the epitaxial layer when C/Si = 1.

In addition to focusing on growth rate and thickness uniformity, the impact of buffer layer growth on the surface roughness of the epitaxial layer was also thoroughly investigated. Surface roughness serves as a crucial metric for evaluating the quality of the epitaxial layer, directly influencing its electrical performance and reliability. Figure 10 illustrates that, when the C/Si ratio is 1, the differences in maximum and minimum roughness values between samples with and without the buffer layer are minimal. However, it is noteworthy that the sample lacking a buffer layer exhibits a slightly lower minimum roughness value (0.314 nm) compared to the sample with a buffer layer. While this difference may appear insignificant, in the realm of nanoscale epitaxial layer growth, even minor variations can significantly impact the epitaxial layer’s performance.

However, upon further analysis of the median and mean values of the data, an intriguing observation was made. While the sample lacking a buffer layer shows a minor advantage in terms of minimum roughness, the sample with a buffer layer demonstrates superior overall performance in surface roughness. The superior surface roughness exhibited by the sample with a buffer layer implies that the growth of the buffer layer could potentially play a crucial role in enhancing the overall surface quality of the epitaxial layer. Buffer layers serve as an intermediary between the substrate and the epitaxial material, providing a platform for controlled nucleation and growth. As a result, the buffer layer may facilitate the formation of smoother and more uniform epitaxial surfaces by mitigating surface irregularities and defects.

**Figure 11:**
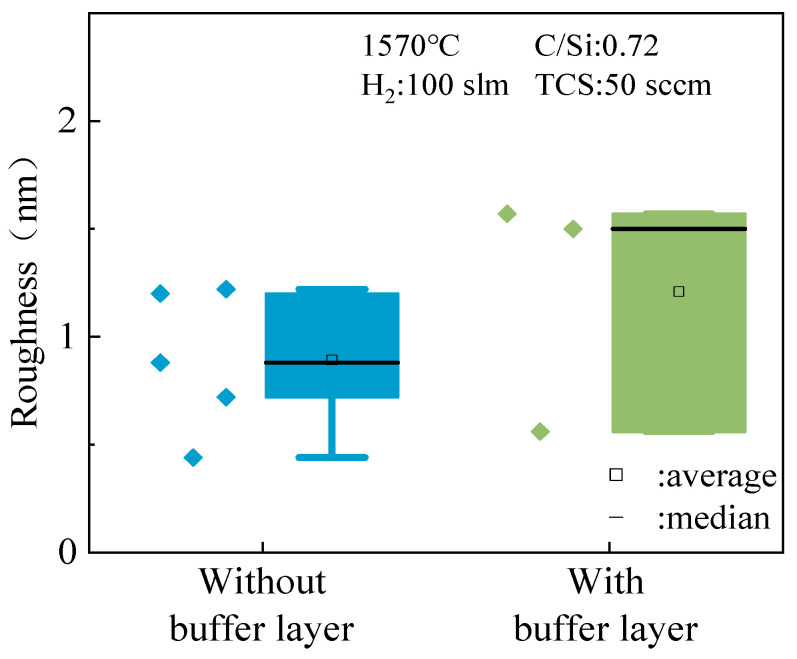
The effect of the buffer layer on the roughness of the epitaxial layer when C/Si = 0.72.

**Figure 12:**
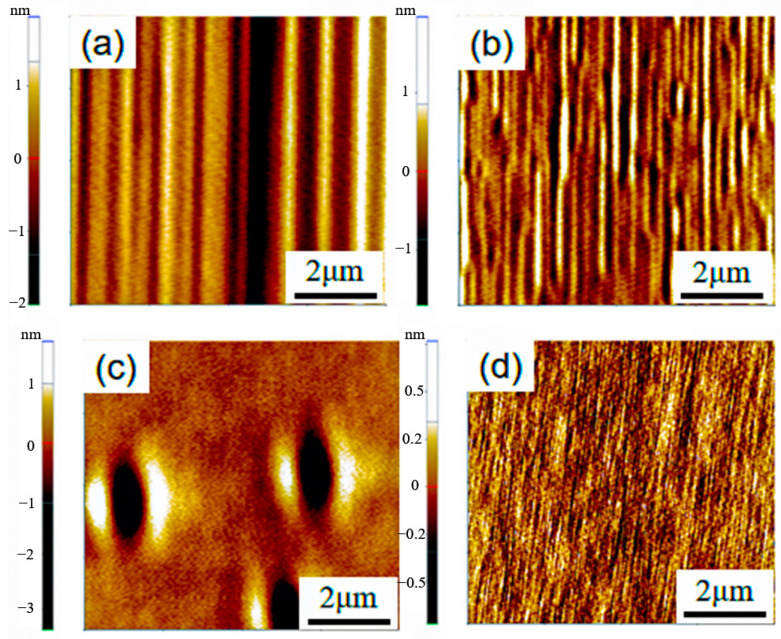
AFM images of samples with and without buffer layers at different C/Si ratios: (**a**) C/Si = 1, without buffer layer; (**b**) C/Si = 1, with buffer layer; (**c**) C/Si = 0.72, without buffer layer; (**d**) C/Si = 0.72, with buffer layer.

Similarly, the impact of buffer layer growth on epitaxial layer roughness was investigated at a C/Si ratio of 0.72. Figure 11 demonstrates that samples without a buffer layer exhibit significantly lower epitaxial layer roughness compared to samples with a buffer layer, as indicated by the data’s volatility, median, and average values. This contrasts with the observations made regarding growth rate and uniformity at a C/Si ratio of 1, emphasizing the close relationship between buffer layer growth and epitaxial layer performance. Varying C/Si ratios result in different effects of the buffer layer, as evidenced by atomic force microscopy (AFM) images provided in Figure 12 for visual comparison. These images support the experimental findings, showcasing distinct surface morphology and roughness between samples with and without a buffer layer.

## 4. Conclusions

This study delved into the impact of three crucial process parameters on the homoepitaxial growth of 4H-SiC on 4H-SiC substrates, focusing on epitaxial layer growth rate (thickness), doping concentration, uniformity, and surface roughness. The comparison between growth conditions using C_3_H_8_ and C_2_H_4_ as carbon sources revealed that while C_3_H_8_ led to a faster growth rate, it resulted in a more dispersed growth rate distribution among samples and poorer surface thickness uniformity. On the other hand, C_2_H_4_, despite yielding a slightly slower growth rate, provided a more stable growth process and significantly improved surface thickness uniformity. Furthermore, the C_2_H_4_-grown epitaxial layer exhibited lower doping concentration but better doping uniformity. Analysis of surface morphology indicated that the epitaxial layer grown with C_3_H_8_ had lower surface roughness, possibly due to specific characteristics of C_3_H_8_ during growth. Conversely, the epitaxial layer grown with C_2_H_4_ displayed higher surface roughness, potentially linked to the more active chemical properties of C_2_H_4_ at high temperatures, leading to a more complex growth process. Considering these factors, it can be inferred that C_2_H_4_ outperforms C_3_H_8_ in terms of growth stability, doping concentration control, and surface morphology, making it the preferred carbon source for 4H-SiC epitaxial growth.

The impact of growth pressure on the growth of epitaxial layers was investigated in this study. It was observed that as the growth pressure increased, the growth rate of the 4H-SiC epitaxial layer gradually decreased, leading to a deterioration in thickness uniformity. Furthermore, both the doping concentration and its uniformity were negatively affected by the increase in pressure. Surface morphology also exhibited changes, with surface roughness initially decreasing and then increasing with varying pressure. These variations are likely attributed to the effects of growth pressure on species transport, reaction kinetics, and substrate surface conditions. Therefore, when optimizing the growth of 4H-SiC epitaxial layers, it is essential to consider the influence of growth pressure comprehensively and fine-tune growth conditions accordingly. It is important to acknowledge that the experimental results may have been influenced by sporadic factors due to the limited number of samples used. To ensure the accuracy and reliability of the conclusions drawn, further experimental verifications are warranted in future research.

Finally, to delve deeper into the impact of buffer layer growth on the 4H-SiC epitaxial layer, comparative experiments were conducted with C/Si ratios of 0.72 and 1. The experimental findings indicate that a C/Si ratio of 1 leads to a reduction in the growth rate and thickness uniformity of the epitaxial layer when the buffer layer grows. Conversely, adjusting the C/Si ratio to 0.72 results in a positive influence of buffer layer growth on the quality of the epitaxial layer and enhances its growth process. Moreover, the research reveals varying trends in the effect of buffer layer growth on the surface roughness of the epitaxial layer based on different C/Si ratio conditions. Specifically, under a C/Si ratio of 1, the surface roughness of the sample with buffer layer growth exhibits superior overall performance, whereas, under a C/Si ratio of 0.72, the sample grown without a buffer layer showcases better surface roughness. Our findings show that the buffer layer grown under different C/Si ratios has completely different effects on the growth rate, uniformity, and roughness of the epitaxial layer. It has been explained that the decision on whether to grow the buffer layer depends on the specific production requirements and cannot be generalized. The next step can be to further study the impact of buffer layer growth under other C/Si ratio conditions for comparison.

## Data Availability

The data that support the findings of this study are available from the corresponding authors, X.Z. and X.L., upon reasonable request.
